# Adipose-specific lipin1 overexpression in mice protects against alcohol-induced liver injury

**DOI:** 10.1038/s41598-017-18837-2

**Published:** 2018-01-11

**Authors:** Wenliang Zhang, Wei Zhong, Qian Sun, Xinguo Sun, Zhanxiang Zhou

**Affiliations:** 1 Center for Translational Biomedical Research, University of North Carolina at Greensboro, North Carolina Research Campus, Kannapolis, NC 28081 USA; 2 Department of Nutrition, University of North Carolina at Greensboro, North Carolina Research Campus, Kannapolis, NC 28081 USA

## Abstract

Excessive fatty acid release from the white adipose tissue (WAT) contributes to the development of alcoholic liver disease (ALD). Lipin1 (LPIN1), as a co-regulator of DNA-bound transcription factors and a phosphatidic acid (PA) phosphatase (PAP) enzyme that dephosphorylates PA to form diacylglycerol (DAG), is dramatically reduced by alcohol in the WAT. This study aimed at determining the role of adipose LPIN1 in alcohol-induced lipodystrophy and the development of ALD. Transgenic mice overexpressing LPIN1 in adipose tissue (LPIN1-Tg) and wild type (WT) mice were fed a Lieber-DeCarli alcohol or isocaloric maltose dextrin control liquid diet for 8 weeks. Alcohol feeding to WT mice resulted in significant liver damage, which was significantly alleviated in the LPIN1-Tg mice. Alcohol feeding significantly reduced epididymal WAT (EWAT) mass, inhibited lipogenesis, and increased lipolysis in WT mice, which were attenuated in the LPIN1-Tg mice. LPIN1 overexpression also partially reversed alcohol-reduced plasma leptin levels. In WT mice, alcohol feeding induced hepatic lipid accumulation and down-regulation of beta-oxidation genes, which were dramatically alleviated in the LPIN1-Tg mice. LPIN1 overexpression also significantly attenuated alcohol-induced hepatic ER stress. These results suggest that overexpression of LPIN1 in adipose tissue restores WAT lipid storage function and secretive function to alleviate alcohol-induced liver injury.

## Introduction

Excessive alcohol consumption has been shown to disrupt lipid homeostasis in the liver and is associated with the development of liver diseases, including steatosis, steatohepatitis, fibrosis, and cirrhosis^1–3^. Excessive alcohol consumption also causes lipid dyshomeostasis at the adipose-liver axis, reducing lipid storage in white adipose tissue (WAT) and decreasing adipose mass, namely lipodystrophy^4–6^. As a major energy storage organ, WAT plays several key roles in mammalian physiology including storage excess energy in the form of triglycerides under positive energy balance conditions and releasing energy in the form of fatty acids under negative energy balance conditions. We and other groups have reported that adipose dysfunction impacts hepatic lipid metabolism^6–8^. WAT also controls energy homeostasis and metabolism through secretion of adipokines such as adiponectin and leptin^9,10^. Leptin is a hormone secreted by adipocytes that plays a pivotal role in the regulation of glucose and lipid metabolism^11^. It has been demonstrated that leptin stimulates triglyceride depletion in WAT and leptin levels are strongly correlated with both percentage fat mass and body mass index^12^.

The lipin protein family, consisting of three members, has been shown to have important roles in a variety of biological processes. The founding member of the family, Lipin1 (LPIN1), was identified in the fatty liver dystrophy mouse model in 2001^13^. LPIN1, highly expressed in adipocytes, works as a phosphatidic acid (PA) phosphatase (PAP) enzyme that dephosphorylates PA to form diacylglycerol (DAG), which is the penultimate step in triglyceride (TG) synthesis^14^. It has been shown that LPIN1 accounts for all of the PAP activity in adipose tissue and skeletal muscle^15^. On the other hand, LPIN1 also works as a co-regulator of DNA-bound transcription factors to translocate to the nucleus and regulate metabolism^14^. It has been shown that LPIN1 directly interacts with peroxisome proliferator-activated receptor α (PPARα) and PPARγ coactivator 1α (PGC-1α) to modulate fatty acid oxidation gene expression^16^. LPIN1 also represses the activity of nuclear factor of activated T cells c4 (NFATc4) to inhibit cytokine production in adipocytes^17^. Our group has reported that LPIN1 was dramatically reduced by alcohol in WAT.

Although various functions of LPIN1 have been elucidated, the role of LPIN1 especially adipose LPIN1 in ethanol-induced lipolysis, hepatosteatosis, ER stress, inflammation, and hepatocyte apoptosis needs to be further explored. The present study was designed to determine the role of LPIN1 in the pathogenesis of alcohol-induced lipodystrophy and the development of ALD.

## Results

### Adipose-specific LPIN1 overexpression inhibits alcohol-induced lipolysis and promotes lipogenesis

**Figure 1:**
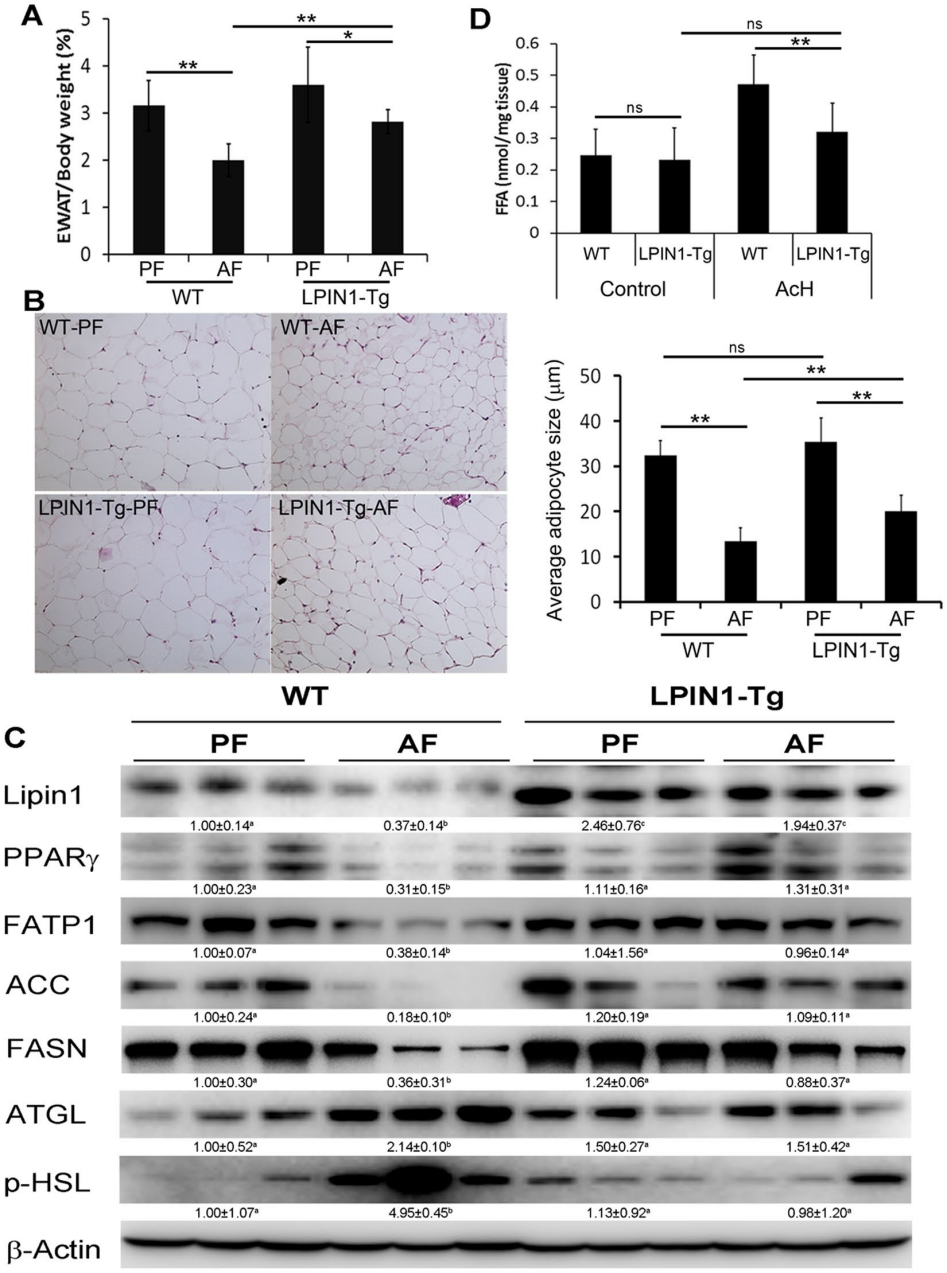
LPIN1 overexpression in adipose tissue restored WAT’s lipid storage function. WT and LPIN1-Tg mice were fed liquid diets containing alcohol (AF) or maltose dextrin (PF) for 8 weeks. (**A**) Ratio of EWAT/Body weight. (**B**) Sizes of adipocytes. (**C**) Levels of proteins involved in lipogenesis and lipolysis. (**D**) FFA release. Data are expressed as means ± SD (n = 8). *P < 0.05; ***P < 0.001; ns, not significant.

EWAT masses of WT mice were significantly reduced by chronic alcohol feeding, adipose-specific LPIN1 overexpression significantly alleviated alcohol-induced reduction of EWAT masses (Fig. 1A). Alcohol feeding significantly decreased EWAT adipocyte size, from 32.44 ± 3.24 µm in WT-PF mice to 13.44 ± 2.99 µm in WT-AF mice. In contrast, adipose-specific LPIN1 overexpression significantly ameliorated alcohol-induced decrease of EWAT adipocyte size (from 35.39 ± 5.33 µm in LPIN1-Tg-PF mice to 20.11 ± 3.46 µm in LPIN1-Tg-AF mice) (Fig. 1B). Immunoblot results showed that in WT mice, alcohol feeding significantly reduced lipogenic genes or regulators, including PPARγ, ACC, FASN, LPIN1, and FATP1, but induced the expression of lipases including p-HSL and ATGL. Adipose-specific LPIN1 overexpression reversed alcohol-reduced expression of lipogenic genes or regulators and alcohol-induced expression of p-HSL and ATGL (Fig. 1C). *Ex vivo* study showed that adipose-specific LPIN1 overexpression significantly inhibited acetaldehyde-induced release of FFAs from EWAT (Fig. 1D).

### Both phosphatidic acid phosphatase and co-regulator of DNA-bound transcription factors functions of LPIN1 contribute to its inhibition of lipolysis

**Figure 2:**
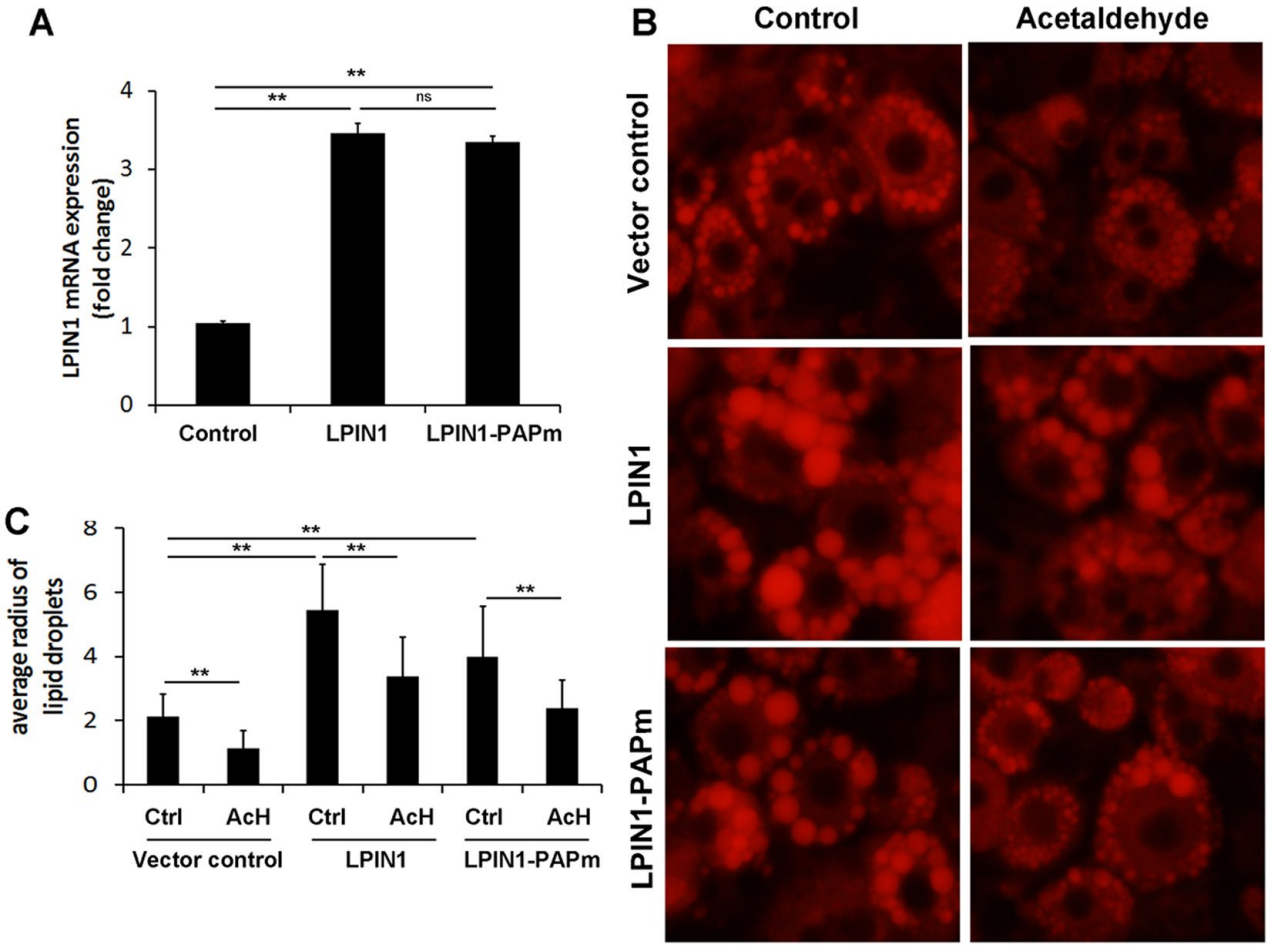
Both phosphatidic acid phosphatase and co-regulator of DNA-bound transcription factors functions of LPIN1 contribute to its inhibition of lipolysis. T3-L1 cells were transfected with retrovirus harboring human LPIN1β-GFP, LPIN1β-PAPm-GFP, or GFP, challenged with acetaldehyde (100 μM) for 3 days. (**A**) qPCR for LPIN1 expression. (**B**) Nile red staining of neutral lipids. (**C**) Quantification of lipid droplets. Data are expressed as means ± SD (n = 3 in A, n = 50 in (**C**)). *P < 0.05; ***P < 0.001; ns, not significant.

RT-PCR results showed that human LPIN1β-GFP and catalytically dead human LPIN1β PAP mutant (LPIN1β-PAPm-GFP) were successfully overexpressed in 3T3-L1 cells (Fig. 2A). Transfected 3T3-L1 cells were challenged with acetaldehyde at a concentration of 100 µmol/L. Nile red staining showed that overexpression of LPIN1β-PAPm-GFP resulted in elevated lipid storage compared with that of adipocytes transfected with GFP, while overexpression of LPIN1β-GFP resulted in further elevation of lipid storage than that of adipocytes transfected with LPIN1β-PAPm-GFP. Administration of acetaldehyde resulted in adipocyte lipolysis, which was significantly alleviated by overexpression of either LPIN1β-PAPm-GFP or LPIN1β-GFP (Fig. 2B,C).

### Adipose-specific LPIN1 overexpression alleviates alcohol-induced liver injury

**Figure 3:**
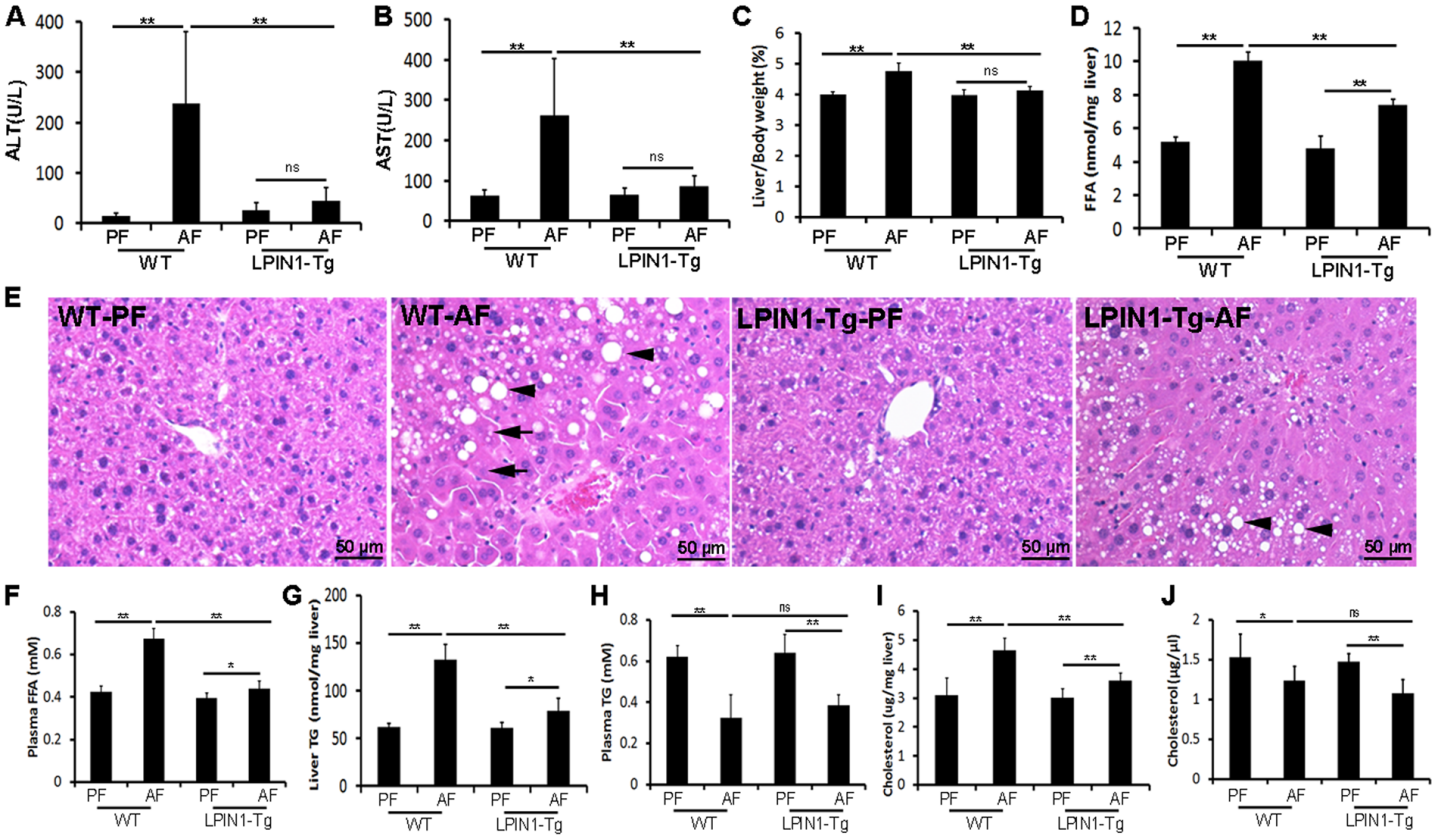
LPIN1 overexpression in adipose tissue alleviated alcohol-induced liver injury. WT and LPIN1-Tg mice were fed liquid diets containing alcohol (AF) or maltose dextrin (PF) for 8 weeks. (**A**) Plasma ALT levels. (**B**) Plasma AST levels. (**C**) Ratio of Liver/Body weight. (**D**) Liver FFA concentrations. (**E**) Liver histopathological changes shown by H&E staining. (**F**) Plasma FFA concentrations. (**G**) Liver TG contents. (**H**) Plasma TG contents. (**I**) Liver cholesterol concentrations. (**J**) Plasma cholesterol concentrations. Data are expressed as means ± SD (n = 8). *P < 0.05; ***P < 0.001; ns, not significant.

Adipose-specific LPIN1 overexpression not only attenuated alcohol feeding-induced elevation of serum ALT and AST (Fig. 3A and B), but also normalized ethanol-increased ratios of liver weight to body weight (Fig. 3C). Hematoxylin and eosin (H&E) staining revealed that 8 weeks of alcohol feeding caused formation of lipid droplets (arrowheads) and necrosis (arrows) in the livers of WT mice, which were alleviated by adipose-specific LPIN1 overexpression (Fig. 3E). In addition, alcohol feeding also significantly elevated levels of both liver FFA and plasma FFA in WT mice, which were significantly alleviated by adipose-specific LPIN1 overexpression (Fig. 3D and F). Adipose-specific LPIN1 overexpression also diminished ethanol-elevated liver TG levels (Fig. 3G), whereas showed no significant effect on ethanol-decreased plasma TG levels (Fig. 3H). Ethanol-elevated liver cholesterol levels were significantly decreased by adipose-specific LPIN1 overexpression (Fig. 3I), but ethanol-decreased plasma cholesterol levels were not significantly affected by adipose-specific LPIN1 overexpression (Fig. 3J).

### Adipose-specific LPIN1 overexpression restores alcohol-reduced fatty acid oxidation and attenuates alcohol-induced ER stress

**Figure 4:**
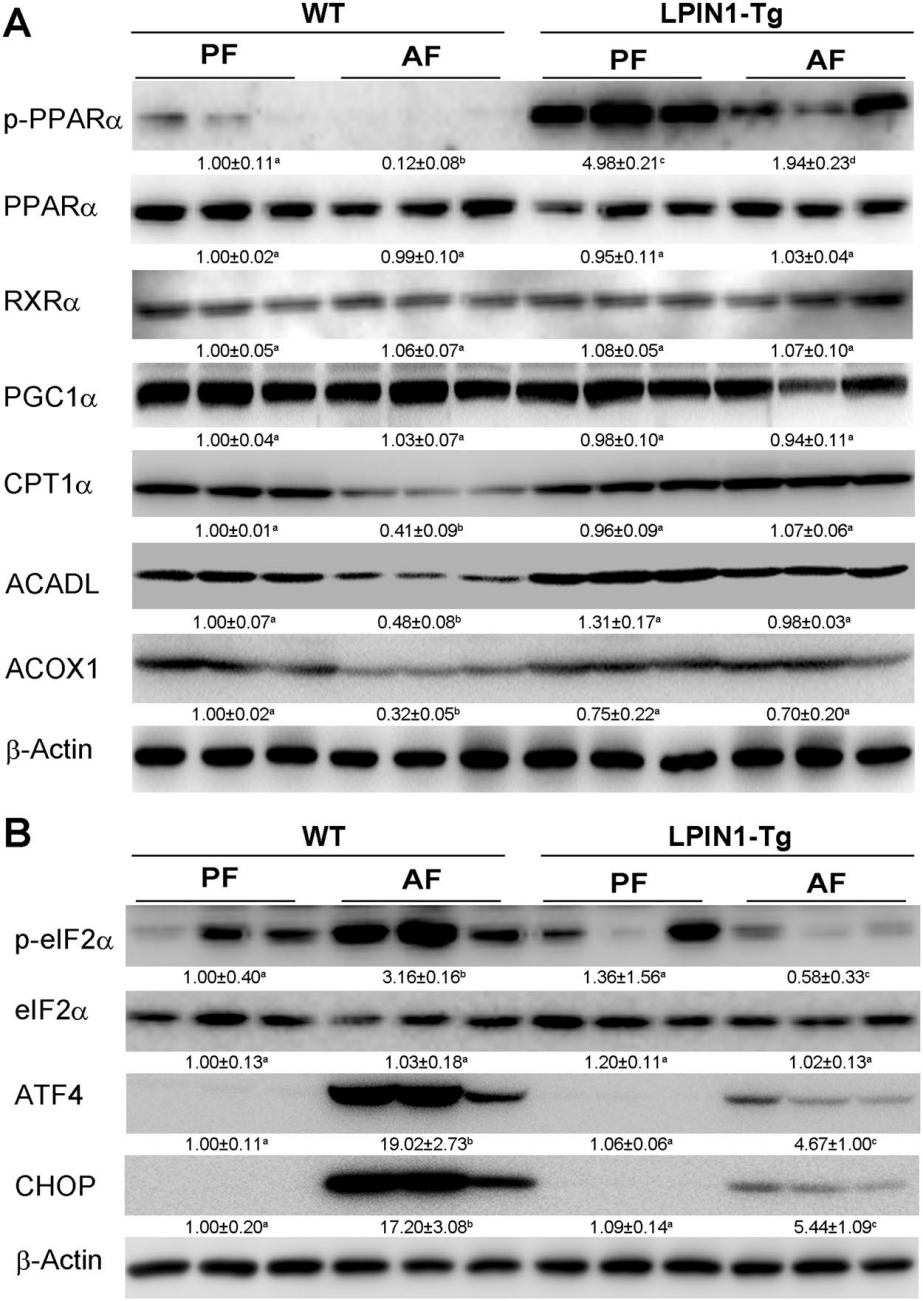
LPIN1 overexpression in adipose tissue alleviated alcohol-suppressed β-oxidation and attenuated alcohol-induced ER stress. WT and LPIN1-Tg mice were fed liquid diets containing alcohol (AF) or maltose dextrin (PF) for 8 weeks. (**A**) Levels of proteins involved in β-oxidation were measured by western blot. (**B**) Levels of proteins involved in ER stress were measured by western blot. Proteins levels were quantitated by NIH image J. All values are denoted as means ± SD (n = 3). Significant differences were indicated by different letters (ANOVA, *P* < 0.05).

Immunoblot results showed that in the livers of WT mice, alcohol feeding significantly reduced the expression of phosphor-PPARα, a transcription factor of lipid metabolism, and the expression of fatty acid oxidation related proteins, including CPT1α, ACOX1, and ACADL, which were reversed by adipose-specific LPIN1 overexpression. Although Adipose-specific LPIN1 overexpression showed no significant effect on the expression of RXRα and PGC-1α (Fig. 4A), it significantly reduced alcohol-induced ER stress markers, including p-eIF2α, ATF4, and CHOP (Fig. 4B).

### Adipose-specific LPIN1 overexpression attenuates alcohol-reduced leptin level to alleviate alcohol-reduced fatty acid oxidation, attenuate alcohol-induced ER stress and protect against alcohol-induced hepatocyte apoptosis

**Figure 5:**
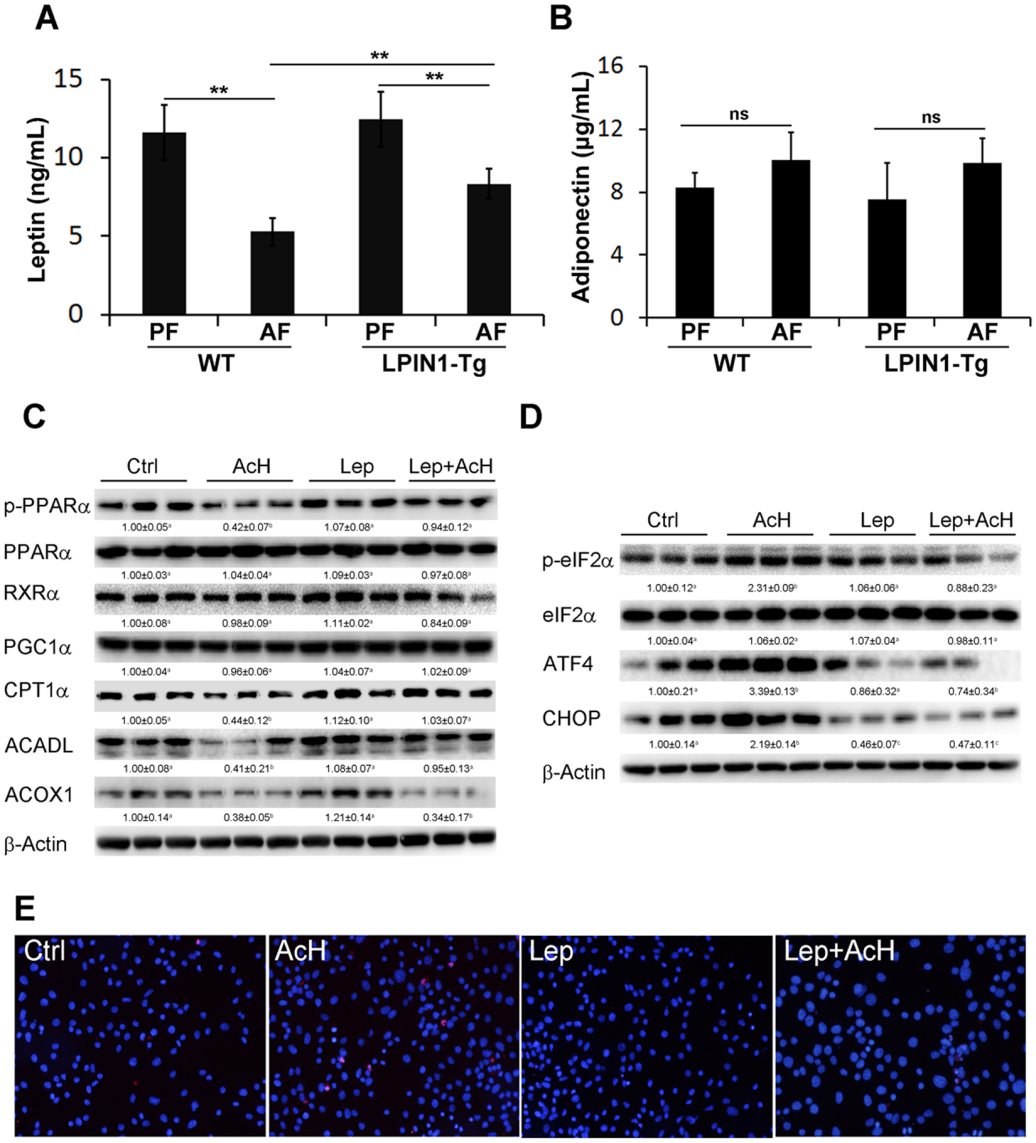
LPIN1 overexpression in adipose tissue alleviated alcohol-induced decrease of leptin level and administration of leptin abolished acetaldehyde-induced ER stress, suppression of β-oxidation, and acetaldehyde-induced apoptosis. WT and LPIN1-Tg mice were fed liquid diets containing alcohol (AF) or maltose dextrin (PF) for 8 weeks. (**A**) Plasma leptin levels. (**B**) Plasma adiponectin levels. AML12 cells were challenged with acetaldehyde (100 μM) for 3 days in the presence or absence of leptin. (**C**) Levels of proteins involved in β-oxidation. (**D**) Levels of proteins involved in ER stress. (**E**) TUNEL staining. Proteins levels were quantitated by NIH image J. All values are denoted as means ± SD (n = 3). Significant differences were indicated by different letters (ANOVA, *P* < 0.05), *P < 0.05; ***P < 0.001; ns, not significant.

Quantitative leptin assay showed that chronic alcohol feeding significantly inhibited the secretion of leptin in WT mice, which was partially reversed by adipose-specific LPIN1 overexpression (Fig. 5A). Adipose-specific LPIN1 overexpression showed no effect on adiponectin secretion (Fig. 5B). Western blots showed that treatment of AML12 cells with 100 µM acetaldehyde inhibited the expression of genes responsible for β-oxidation including p-PPARα, CPT1α, ACADL, and ACOX1 (Fig. 5C), and induced ER stress (Fig. 5D), which were reversed by treatment of cells with leptin (100 µM). TUNEL staining of hepatocytes showed that challenge of AML12 cells with 100 µM acetaldehyde resulted in significant apoptosis, which was dramatically alleviated by administration of leptin (Fig. 5E).

### Adipose-specific LPIN1 overexpression attenuates alcohol-induced adipose tissue inflammation

**Figure 6:**
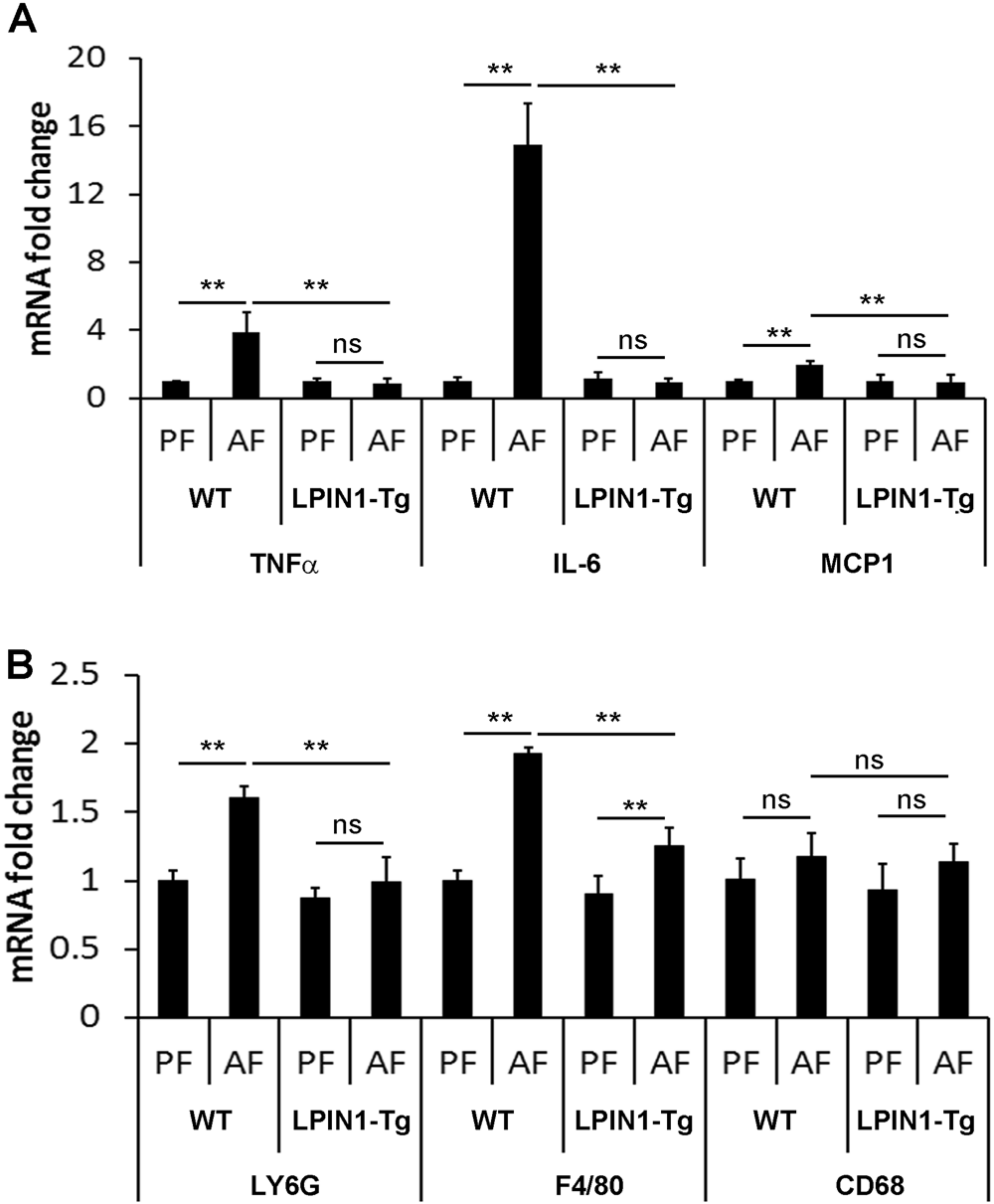
Effect LPIN1 overexpression in adipose tissue on inflammatory cell infiltration and inflammatory chemokine expression. WT and LPIN1-Tg mice were fed liquid diets containing alcohol (AF) or maltose dextrin (PF) for 8 weeks. (**A**) The expression changes of inflammatory chemokines. (**B**) The expression changes of markers of inflammatory cell infiltration. Data are expressed as means ± SD (n = 3). **P < 0.01; ***P < 0.001; ns, not significant.

RT-PCR results showed that pro-inflammatory cytokines and chemokines including TNFα, IL-6, and MCP1 were significantly induced by alcohol exposure in the EWAT of WT mice, which were significantly attenuated in the EWAT of LPIN1-Tg mice (Fig. 6A). Inflammatory infiltration markers including Ly6G and F4/80 were significantly upregulated by alcohol exposure in the EWAT of WT mice, which was attenuated by adipose-specific LPIN1 overexpression (Fig. 6B). Alcohol feeding or LPIN1 overexpression showed no effect on CD68 (Fig. 6B).

## Discussion

The role of adipose-LPIN1 in alcohol-induced liver injury was investigated in this study. Data showed that adipose-specific LPIN1 overexpression protected mice against alcohol-induced liver injury via: (1) promoting alcohol-inhibited de novo lipogenesis but inhibiting alcohol-promoted lipolysis in white adipose tissue to restore alcohol-decreased size of adipocytes and the ratio of WAT to body weight, (2) promoting alcohol-inhibited β-oxidation in the liver to reduce ER stress, (3) increasing the release the leptin to alleviate alcohol-induced liver apoptosis, and (4) alleviating inflammation.

WAT is a major energy storage organ which stores energy in the form of triglycerides. The function and mass of WAT is tightly related to the synthesis and degradation of triglycerides. Excess free fatty acid flux or ectopic fat accumulation, has been considered as a key risk factor in the development of a number of chronic diseases, including type 2 diabetes and cardiovascular disease^18^. Excess adipose tissue lipolysis has also been shown to induce reverse-transportation of FFA from adipose tissue to liver and result in fatty liver^6^. Highly expressed in adipocytes, LPIN1 not only catalyzes the penultimate step in TG synthesis, i.e. from PA to DAG, but also works as a co-regulator of DNA-bound transcription factors to translocate to the nucleus and regulate metabolism^14^. This current study showed that in WAT of WT mice, alcohol feeding not only inhibited de novo lipogenesis but also promoted lipolysis, leading to lipid dyshomeostasis. In contrast, adipose-specific LPIN1 overexpression significantly alleviated alcohol-inhibited de novo lipogenesis and alcohol-induced lipolysis, thereby restored adipocyte size and the ratio of WAT to body weight and improved the energy storage function of WAT shown by reduced FFA release from WAT under acetaldehyde challenge (Fig. 1).

Our previous study has demonstrated that WAT expresses high levels of ALDH isoforms, including ALDH1A1, ALDH2 and ALDH3A1, but low level of ADH and CYP2E1, suggesting that WAT plays a role in aldehyde catabolism rather than ethanol catabolism. Indeed, we further showed acetaldehyde exposure, rather than ethanol exposure, suppressed lipogenetic enzymes including LPIN in 3T3-L1 cells^8^. In order to dissect the two functions of LPIN1, LPIN1β-GFP and catalytically dead LPIN1β PAP mutant (LPIN1β-PAPm-GFP) were successfully overexpressed in 3T3-L1 cells. The genetically modified 3T3-L1 cells were exposed to acetaldehyde to mimic alcohol exposure condition. Nile red staining results showed that overexpression of LPIN1β-PAPm-GFP resulted in elevated lipid storage, while overexpression of LPIN1β-GFP resulted in further elevation of lipid storage. Administration of acetaldehyde resulted in significant adipocyte lipolysis, which was alleviated by overexpression of either LPIN1β-PAPm-GFP or LPIN1β-GFP (Fig. 2). All the data suggested that both PAP and co-regulator of DNA-bound transcription factors functions contribute to enhanced lipid storage capacity. Previous study showed that LPIN1 functions as a key regulator of PPARγ activity through its ability to release co-repressors and recruit co-activators^19^. We observed that adipose specific LPIN1 overexpression reversed alcohol-suppressed PPARγ expression and other lipogenic gene expression including FASN and ACC, suggesting that LPIN1 might also regulate PPARγ expression directly through its co-regulator of DNA-bound transcription factors function.

Fatty acid β-oxidation is the catabolic process to broken down fatty acid molecules to generate acetyl-CoA in the mitochondria of eukaryotes. β-oxidation also occurs in peroxisomes when the fatty acid chains are too long to be handled by the mitochondria^20^. The enzyme catalyzing the first step of mitochondrial- and peroxisomal-β-oxidation is acyl-CoA dehydrogenases, which include very-long-chain, long-chain, medium-chain, and short-chain acyl-CoA dehydrogenases, and acyl-CoA oxidase (ACOX), respectively^20,21^. Alcohol feeding significantly inhibits ACADL and ACOX1, the enzymes responsible for both mitochondrial- and peroxisomal-β-oxidation. It has been shown that PPARα not only controls gene expression levels of the rate-limiting enzymes of peroxisomal β-oxidation, including ACOX1, but also directly controls ACADL expression levels to regulate the critical reaction of mitochondrial β-oxidation^22–24^. Our results showed that alcohol feeding significantly suppressed the expression of transcription factor phospho-PPARα and its targets genes including ACADL and ACOX1. Alcohol-suppressed fatty acid β-oxidation and abovementioned alcohol-enhanced adipose tissue lipolysis together led to hepatic ER stress shown by up-regulated phospho-eIF2α, ATF4 and CHOP. In contrast, adipose specific LPIN1 overexpression reversed alcohol-suppressed transcription factors and essential enzymes involved in fatty acid β-oxidation, improved the lipid storage function of WAT, and alleviated hepatic ER stress (Fig. 4). Previous studies showed that PGC1α was a coactivator of PPARα and RXR^25–27^, and PPARα binds as heterodimer with the retinoid X receptor (RXRα) to a specific DNA sequence termed the peroxisome proliferator response element (PPRE)^28,29^. However, in the present study, we did not see any effect of alcohol feeding on the expression of RXRα or PGC-1α either in WT mice or in LPIN1 transgenic mice.

Adipose tissue has recently recognized as endocrine tissue by producing multiple adipokines including leptin and adiponectin^30–34^. Decreased adiponectin levels are associated with dyslipidemia and levels of plasma adiponectin have been negatively correlated with triglycerides^35–37^. Abundantly expressed in adipose tissue, specifically adipocytes, leptin is involved in the regulation of energy homeostasis via inhibition of appetite and food intake and stimulation of energy expenditure^31^. This led us to determine the levels of leptin and adiponectin. As shown in Fig. 5, adipose specific LPIN1 overexpression significantly alleviated alcohol-reduced leptin level. Neither alcohol nor LPIN1 overexpression affected adiponectin level. Treatment of hepatocyte AML12 cells with the metabolite of alcohol, acetaldehyde, significantly inhibited transcription factors and enzymes involved in fatty acid β-oxidation, including phospho-PPARα, CPT1α, ACADL, and ACOX1 and resulted in hepatic ER stress shown by up-regulated phosphor-eIF2α, ATF4, and CHOP, which were abolished by administration of leptin. Moreover, administration of leptin significantly attenuated acetaldehyde-induced AML12 cell apoptosis. All the results suggested that leptin directly affects fatty acid β-oxidation and subsequent lipotoxicity and could be used for treatment of obesity and other diseases associated with hyperlipidemia (e.g. hypothyroidism, diabetes, renal insufficiency, etc.)^38^.

Inflammation has been involved in a variety of diseases including ALD and NAFLD^39–41^. Our group has showed that MCP1, IP-10, and KC play important roles in the animal model of ALD and alcohol feeding significantly induced inflammatory cell infiltration^39^. In this study, our data suggested that restoration of WAT lipid storage function and leptin secretion by overexpression of LPIN1 in adipose tissue significantly alleviated alcohol feeding-induced inflammatory cytokine production and inflammatory cell infiltration, which might shed light on the treatment of inflammatory diseases.

## Conclusions

In short, the present study demonstrated that chronic alcohol feeding resulted in adipose tissue lipodystrophy and subsequent liver injury including ER stress, apoptosis, and inflammation. Overexpression of LPIN1 in adipose tissue protected mice against alcohol-induced liver injury through restoration of WAT lipid storage function and leptin secretion.

## Materials and Methods

### Generation of LPIN1 transgenic mice

All experiments were performed in accordance with relevant guidelines and regulations of North Carolina Research Campus. pBS aP2 promoter (5.4bk) polyA plasmid (#11424) and pLKO-puro FLAG wildtype, catalytic active LPIN1 plasmid (#32010) were obtained from Addgene (Cambridge, MA). The mouse LPIN1 coding sequence (without FLAG tag) was amplified with primers Lipin-F (5′-AGTTCTAGAGCGGCCACCATGAATTACGTGGGGCAGCTG-3′) and Lipin-R (5′-TCGTATTACGCGGCCGCTCAAGCTGAGGCTGA-3′). The amplified LPIN1 cDNA was cloned into the NotI site of the aP2 vector and sequence verified. The resulting AP2-LPIN1-SV40pA transgene was excised with KpnI and SacII and gel purified. The purified transgene was microinjected into C57BL/6J zygotes, which were implanted into surrogate females for transgenic founder production. Pups born from the microinjected embryos were genotyped with primers Lipin-SqF2 (5′- GCAGTTTGTGAACGAGGAGGATC-3′ and Lipin-SqR2 (5′- ATTGCCAACCTTGACCACGAG-3′) to get transgenic founders.

### Animals and alcohol feeding

The animal protocol was approved by the Institutional Animal Care and Use Committee of the North Carolina Research Campus. Ten-week-old male wild type (WT) and LPIN1-Tg mice were pair-fed a modified Lieber-DeCarli alcohol or isocaloric maltose dextrin control liquid diet (Dyets, Bethlehem, PA) for 8 weeks (n = 8) with a stepwised feeding procedure, as described previously^42^. Briefly, the ethanol content (%, w/v) in the diet was started at 3.0 and gradually increased to 4.4. The amount of food given to the pair-fed mice was the same as what the alcohol-fed mice consumed in the previous day. At the end of 8-week feeding, mice were anesthetized with inhalation of isoflurane and tissues were collected.

### Cell culture

Mouse AML12 hepatocytes (ATCC, Rockville, MD, USA) were cultured in Dulbecco’s modified eagle medium: Nutrient mixture F-12 (DMEM/F12, Thermo fisher scientific, Waltham, MA, USA) containing 10% fetal bovine serum (FBS; Atlanta Biologicals, Lawrenceville, GA), 100 U/ml penicillin and 100 μg/ml streptomycin (Invitrogen), at 37 °C in a humidified atmosphere of 5% CO2. 3T3-L1 fibroblast cells (ATCC, Rockville, MD, USA) were cultured and differentiated as previously described^8,43^.

### Histopathological analysis

Plasma alanine aminotransferase (ALT) and aspartate aminotransferase (AST) activity was colorimetrically measured using Infinity kits (Thermo Scientific, Waltham, MA). Liver tissue paraffin sections were prepared and stained with hematoxylin and eosin (H&E). Lipid quantitative assays were conducted by measuring the concentrations of hepatic triglyceride and FFA in the liver tissues using kits from BioVision (Milpitas, CA). Epididymal WAT (EWAT) paraffin sections were also prepared and stained with hematoxylin and eosin (H&E).

### Quantitative assay of leptin

Levels of plasma leptin were measured with a commercial kit according to manufacturer’s instructions (Thermo Scientific, Waltham, MA).

### Immunoblot analysis

Proteins from livers or cells were extracted using 10% Nonidet P-40 lysis buffer supplemented with protease inhibitor and phosphatase inhibitor (Sigma-Aldrich, St. Louis, MO). Aliquots containing 50 µg of proteins were loaded onto 8–12% SDS-PAGE, trans-blotted onto PVDF membrane, blocked with 5% nonfat milk in Tris-buffered saline solution with 0.1% Tween-20 for 1 hour at room temperature, and incubated with anti-LPIN1 (Abcam, Cambridge, MA), activating transcription factor 4 (ATF4), PGC1α (Novus Biologicals, Littleton, CO), phospo-peroxisome proliferator-activated receptor α (p-PPARα), PPARα (Thermo Scientific, Waltham, MA), fatty acid transport protein 1 (FATP1), C/EBP homologous protein (CHOP), β-actin (Santa Cruz Biotechnology, Inc., Santa Cruz, CA), fatty acid synthase (FASN), long chain acyl CoA dehydrogenase (ACADL), acyl CoA oxidase 1 (ACOX1), carnitine palmitoyltransferase 1A (CPT1α) (Proteintech, Rosemont, IL), acetyl-CoA carboxylase (ACC), phospho-hormone-sensitive lipase (p-HSL), adipose triglyceride lipase (ATGL), eukaryotic translation initiation factor 2α (eIF2α), phospho-eIF2α, RXRα, PPARγ (peroxisome proliferator-activated receptor-γ) antibody (Cell Signaling Technology, Danvers, MA), respectively. Membranes were washed and incubated with horseradish peroxidase–conjugated secondary antibodies (Thermo Scientific, Rockford, IL, USA). Bound complexes were detected via enhanced chemiluminescence (GE Healthcare, Piscataway, NJ, USA). Bands were quantified, and the ratio to β-actin was calculated and given as fold changes, setting the values of pair-fed or control at 1.

### qPCR analysis

Total RNA was isolated from 3T3-L1 cells or EWAT with TRIzol reagent (Life Technologies, Grand Island, NY) and reverse transcribed with TaqMan Reverse Transcription Reagents (Applied Biosystems, Carlsbad, CA). The gene expression was measured in triplicate with SYBR green PCR Master Mix (Qiagen, Valencia, CA) by the comparative cycle threshold method using 7500 real time PCR system (Applied Biosystems). The primers (Table 1) were purchased from Integrated DNA Technologies (Coralville, IA). Data were normalized to 18s rRNA and expressed as relative changes setting the values of control as one.

**Table 1:** Primer sequences used for qPCR analysis.

Gene	Forward/Reverse (5′–3′)
Ly6G	CCACTCCTCTCTAGGACTTTCA/ACCTTGGAATACTGCCTCTTTC
F4/80	TACCACTTGCCCAGCTTATG /GGGCCTTGAAAGTTGGTTTG
CD68	ATTGAGGAAGGAACTGGTGTAG/CCTCTGTTCCTTGGGCTATAAG
MCP-1	GTCCCTGTCATGCTTCTGG/GCTCTCCAGCCTACTCATTG
TNFα	CTTCTGTCTACTGAACTTCGGG/CAGGCTTGTCACTCGAATTTTG
IL-6	TAGTCCTTCCTACCCCAATTTCC/TTGGTCCTTAGCCACTCCTTC
LPIN1	GGTTTTCAGTGTCACCACGC/CAAGGGTGGGCAAAATGTGG
18s rRNA	GTAACCCGTTGAACCCCATT/CCATCCAATCGGTAGTAGCG

### Assessment of WAT lipolysis by *ex vivo* explants culture

Adipose tissue explants were obtained from the EWAT, cut into small pieces, and placed in a culture plate with pre-warmed PBS containing penicillin (100 U/ml) and streptomycin (100 mg/ml) to remove connective tissue and blood vessels. Explants (20 mg) were transferred to 24-well plates and cultured in 200 µl DMEM with L-glutamine (2 mM), penicillin (50 U/ml), streptomycin (50 mg/ml), and 2% fatty acid-free bovine serum albumin for 2 hours at 37 °C in the presence of absence of acetaldehyde (100 uM). FFA released in the culture medium was measured by a FFA Quantification Kit (BioVision, Mountain View, CA).

### Effect of LPIN1 on lipid storage capability of 3TL-L1 cells

One day post-differentiation, 3T3-L1 cells were transfected with retroviral infectious media from the Phoenix cultures harboring human LPIN1β-GFP, catalytically dead human LPIN1β PAP mutant (LPIN1β-PAPm-GFP), or GFP (a general gift of Dr. Symeon Siniossoglou, Cambridge Institute for Medical Research, University of Cambridge) as previously described^44–46^. Two days post-transduction, cells were challenged with acetaldehyde (100 μM) for 3 days. Cells were either collected for mRNA measurement or fixed with 4% paraformaldehyde at room temperature for 30 min to process with nile red solution to stain neutral lipids. Fluorescent images were captured by Leica TCS SP5 confocal system (Leica, Wetzlar, Germany).

### Assessment of the effect of leptin on ER stress and β-oxidation

Mouse AML12 hepatocytes (80% confluent) were challenged with acetaldehyde (100 µM, Sigma-Aldrich, St. Louis, MO) for 3 days in the presence or absence of leptin (100 ng/ml, BioVision, Milpitas, CA). Cells were collected and proteins were extracted.

### Terminal deoxynucleotidyl transferase dUTP Nick End Labeling (TUNEL) assay

AML12 cells were routinely cultured in DMEM/F12 (Thermo Scientific, Waltham, MA). TUNEL assay kit (EMD Millipore, Billerica, MA) was used to detect DNA fragmentation^47^. Leptin (100 ng/ml, BioVision, Milpitas, CA), or acetaldehyde (100 µM, Sigma-Aldrich, St. Louis, MO) was used to treat AML12 cells for 3 days. Cell were fixed with 1% paraformaldehyde for 10 min, pretreated with proteinase K and H_2_O_2_, and incubated with the reaction mixture containing terminal deoxynucleotidyl transferase (TdT) and digoxigenin-conjugated dUTP for 1h at 37 °C. The labeled DNA was visualized with Alexa Fluor 594-conjugated anti-digoxigenin antibody (Jackson Immunoresearch, West Grove, PA). DAPI was used for counterstaining.

### Statistical analysis

Data are expressed as mean ± standard deviation (SD). Results were analyzed using the one-sample *t*-test or one-way analysis of variance (ANOVA) followed by Turkey’s HSD. In all tests, P values less than 0.05 were considered statistically significant.
